# A Computational Approach to Justifying Stratifin as a Candidate Diagnostic and Prognostic Biomarker for Pancreatic Cancer

**DOI:** 10.1155/2022/1617989

**Published:** 2022-05-02

**Authors:** Md Roman Mogal, Asadullah Junayed, Md Rashel Mahmod, Sagarika Adhikary Sompa, Suzana Afrin Lima, Newton Kar, TasminaTarin, Marina Khatun, Md Abu Zubair, Md Asaduzzaman Sikder

**Affiliations:** ^1^Department of Biochemistry and Molecular Biology, Mawlana Bhashani Science and Technology University, Tangail 1902, Bangladesh; ^2^Department of Food Technology and Nutritional Science, Mawlana Bhashani Science and Technology University, Tangail 1902, Bangladesh

## Abstract

Pancreatic cancer (PC) is considered a silent killer because it does not show specific symptoms at an early stage. Thus, identifying suitable biomarkers is important to avoid the burden of PC. Stratifin (SFN) encodes the 14-3-3*σ* protein, which is expressed in a tissue-dependent manner and plays a vital role in cell cycle regulation. Thus, SFN could be a promising therapeutic target for several types of cancer. This study was aimed at investigating, using online bioinformatics tools, whether SFN could be used as a diagnostic and prognostic biomarker in PC. SFN expression was explored by utilizing the ONCOMINE, UALCAN, GEPIA2, and GENT2 tools, which revealed that SFN expression is higher in PC than in normal tissues. The clinicopathological analysis using the ULCAN tool showed that the intensity of SFN expression is commensurate with cancer progression. GEPIA2, R2, and OncoLnc revealed a negative correlation between SFN expression and survival probability in PC patients. The ONCOMINE, UCSC Xena, and GEPIA2 tools showed that cofilin 1 is strongly coexpressed with SFN. Moreover, enrichment and network analyses of SFN were performed using the Enrichr and NetworkAnalyst platforms, respectively. Receiver operating characteristic (ROC) curves revealed that tissue-dependent expression of the SFN gene could serve as a diagnostic and prognostic biomarker. However, further wet laboratory studies are necessary to determine the relevance of SFN expression as a biomarker.

## 1. Introduction

The pancreas is a pear-shaped organ located in the abdomen, and it plays an essential role in converting foods to become fuel for body cells. However, in some cases, the growth of the pancreas becomes uncontrollable due to some reasons and thus becomes cancerous. Pancreatic cancer (PC) is one of the deadliest cancers and is the seventh most common cause of cancer-related deaths in both men and women [1]. According to GLOBOCAN, in 2018, the estimated number of PC cases and deaths were 458,918 and 432,242, respectively, corresponding to 2.5% of all new cancer diagnoses and 4.5% of all cancer deaths [1]. PC has become more common in recent decades, and the number of new cases will reach 355,317 by 2040 [2, 3]. PC incidence is 3–4 times greater in developed countries than in developing and poor countries [4]. In the United States, it is estimated that in 2021, approximately 60,430 individuals (31,950 men and 28,480 women) will be diagnosed with PC and approximately 48,220 individuals (25,270 men and 22,950 women) will die of PC [5]. Furthermore, PC is expected to overtake breast cancer as the third leading cause of cancer-related death in the European Union, as in the United States [3, 6].

PC is often difficult to diagnose at an early stage; as a result, the majority of PC cases are diagnosed at an advanced stage, and only 10–20% of cases are surgically treatable [7]. This trend is due to the lack of distinct clinical signs and symptoms, due to a lack of accurate biomarkers, and due to the limited resolution of imaging techniques, resulting in a high mortality rate in PC [7, 8]. The 5-year overall survival rate for PC has remained low at 3% in recent years, as more than half of PC patients are diagnosed at an advanced stage [9, 10]. Compared with the screening programs for other cancers, such as lung, breast, colon, and cervical cancers, those for PC are difficult to implement due to the lack of specificity of a particular test [11]. The most common biomarker that has been approved by the US Food and Drug Administration (FDA) for PC diagnosis is the carbohydrate antigen (CA) 19-9. However, CA has not been considered to be the most effective screening tool due to its low sensitivity and specificity and poor predictive value of 0.5–0.9% in asymptomatic patients [12, 13]. Meanwhile, CA 19-9 expression may increase in other medical conditions, such as acute cholangitis, pancreatitis, obstructive jaundice, and liver cirrhosis [11]. Currently, there are no biomarkers with an adequately high accuracy that could be used to screen sporadic PC; therefore, there is an urgent need to identify biomarkers for PC [14].

Stratifin (SFN) encodes the 14-3-3*σ* protein, which is a member of a highly conserved family of 14-3-3 proteins found in all eukaryotic organisms [15]. SFN was first identified as human mammary epithelial marker 1 before being rediscovered as a key regulator of cell cycle checkpoints [16, 17]. Decreased SFN expression has been found in various cancers, including breast [18], lung [19], liver [20], endometrium [21], head and neck [22, 23], vulva [24], and prostate cancers [25, 26]. Conversely, upregulation of the SFN gene expression has been observed in other cancers, including pancreatic [27–29], colorectal [30], and esophageal squamous cell carcinoma [31]. The expression of the SFN gene varies in different cancers, and it performs a double-edged function [32]. Therefore, the role of SFN expression is likely context dependent. On the basis of its tissue-dependent expression pattern, SFN can be used as a diagnostic and prognostic biomarker in PC. However, there has been insufficient evidence demonstrating that SFN expression can be used as a biomarker for PC.

**Figure 1:**
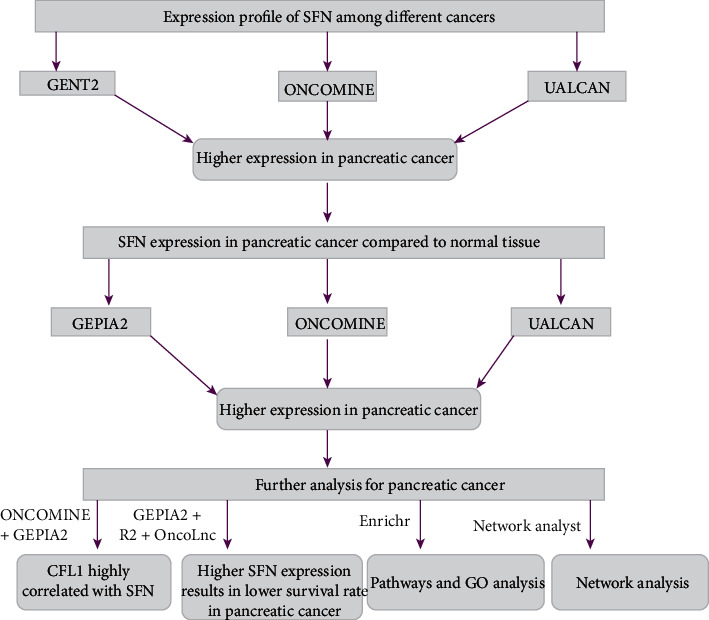
Schematic of the overall workflow in this study.

This study compared the expression pattern of SFN in PC patients and healthy individuals based on data obtained from online databases. Moreover, clinicopathological features, coexpression, prognostic values, gene ontologies, signaling pathways, and network analysis were performed. The workflow for this study is depicted in Figure 1.

## 2. Material and Methods

### 2.1. Investigation of mRNA Expression in Human Cancers

The ONCOMINE (https://http://www.oncomine.org/) tool was used to examine the mRNA expression levels of SFN in different cancers, wherein the threshold level for *P* value, gene rank, and fold change were fixed at 1 × 10^−4^, 10%, and 2, respectively. ONCOMINE is a web-based data-mining platform that is aimed at facilitating the identification of cancer-related genes by analyzing genome-wide expression [33, 34]. The pancancer view for SFN was determined using the UALCAN (http://ualcan.path.uab.edu/) platform. UALCAN is a comprehensive, user-friendly, and interactive web-based resource used to study cancer OMICS data [35]. Then, GENT2 (http://gent2.appex.kr/gent2/) [36] was adopted by using the GPL570 platform (HG_U133_Plus_2) to investigate the SFN expression levels in different types of cancer.

### 2.2. SFN Expression in PC versus Healthy Tissues

We examined the SFN expression in various datasets collected from the ONCOMINE tool. The SFN expression levels under normal conditions were explored in different PC subtypes, such as pancreatic carcinoma, pancreatic adenocarcinoma, and pancreatic ductal adenocarcinoma. Moreover, the SFN expression in PC obtained from the GEPIA2 (http://gepia2.cancer-pku.cn/#index) platform was compared with that in their normal counterpart. GEPIA2 is a web-based platform used for gene expression analysis involving data for tumor and normal samples retrieved from the TCGA and GTEx databases [37]. UALCAN was utilized to obtain SFN expression data in PC and then compared with those in normal tissues.

### 2.3. SFN Expression in relation to Clinicopathological Parameters in PC

The UALCAN web tool with default settings was used to assess the mRNA expression of the SFN gene in PC patients based on their clinicopathological features. In this investigation, SFN expression was analyzed based on clinicopathological parameters, such as cancer stages, race, age, nodal metastasis status, and tumor grade. Only the statistically significant results were taken into account in the analysis.

### 2.4. Association between SFN Expression and Survival Probability in PC Patients

The impact of SFN expression on the survival probability of PC patients was investigated using the GEPIA2, R2 (http://r2platform.com), and OncoLnc (http://www.oncolnc.org/). The R2 genomics platform is a publicly available web-based platform that allows researchers to integrate, analyze, and visualize clinical and genomics data [38]. OncoLnc is an online tool for estimating survival relationships and for accessing clinical data for mRNAs, miRNAs, and lncRNAs (long noncoding RNAs) [39]. The R2 platform was utilized to generate a Kaplan-Meier plot (OS) for the SFN gene against the mixed tumor pancreas Hussain-130-rma-sketch-hugene10t and mixed pancreatic adenocarcinoma Sadanandam-47-MAS5.0-u133p2 datasets by setting the optimum cut-off values. The Kaplan-Meier plot was drawn by splitting the patient population at the median. A *P* < 0.05 was considered significant.

### 2.5. Coexpression Analysis of the SFN Gene in PC Cancer

The SFN gene's coexpression profile in PC was determined, and the corresponding heat map was obtained from the Collisson Pancreas dataset through the ONCOMINE web tool. From this dataset, the cofilin 1 (CFL1) gene was the most positively correlated with SFN expression in PC. To confirm the relationship between SFN and CFL1, we used the TCGA (PAAD) dataset from the UCSC Xena server (https://xenabrowser.net/) [40]. Furthermore, correlation data were obtained from the UCSC Xena server, and a scatter plot was drawn by using ggplot2 [41]. The GEPIA2 was utilized to confirm the positive correlation between SFN and CFL1 transcripts in the PC.

### 2.6. Enrichment Analysis of the SFN Gene

The Enrichr (https://maayanlab.cloud/Enrichr/) web tool was used to extract the gene ontologies and signaling pathways of the SFN gene, as well as the corresponding bar graphs. Enrichr is a user-friendly web-based enrichment analysis tool that graphically presents the collective functions of genes [42, 43]. Gene ontologies were analyzed using GO Biological Process 2018, GO Molecular Process 2018, and GO Cellular Process 2018. Signaling pathways were determined using BioPlanet 2019, Reactome 2016, WikiPathway 2021 Human, KEGG 2021 Human, Biocarta 2016, and Panther 2016.

### 2.7. Evaluation of the SFN Interaction Network

The STRING (https://string-db.org/) database was employed to investigate the interactions of SFN with other proteins. STRING is a database that contains information on direct (physical) and indirect (functional) connections for over 2000 organisms [44]. We also used the GeneMANIA (https://genemania.org/) web platform to create an interaction network of closely linked genes. GeneMANIA is used to predict the function of a gene or gene lists and to identify the physical interaction, genetic interactions, coexpression, pathway, colocalization, and shared protein domain [45].

### 2.8. TF and miRNA Network Analyses

TFs are proteins that regulate gene expression by binding to certain DNA sequences [46], and miRNAs are a type of noncoding RNAs that play crucial functions in gene regulation [47]. TF and miRNA networks were constructed based on the ChEA [48] and TarBase [49] repositories, respectively, using the NetworkAnalyst (https://dev.networkanalyst.ca/NetworkAnalyst/uploads/ListUploadView.xhtml) web platform. NetworkAnalyst is a comprehensive web tool used for gene expression analysis, and it generates visual networks [50].

### 2.9. ROC Curve Analysis of the SFN Gene

For determining the diagnostic and prognostic values of the SFN gene, receiver operating characteristic (ROC) curves were drawn. For this purpose, gene expression data (GSE16515) were retrieved from the Gene Expression Omnibus (GEO) database (https://www.ncbi.nlm.nih.gov/gds), and survival data were obtained from TCGA-PAAD through the OncoLnc (http://www.oncolnc.org/) platform. The ROC curve was plotted, and the area under the ROC curve (AUC) was calculated by exploiting the Statistical Packages for Social Sciences (SPSS for Windows, version 20, IBM Corp., Armonk, New York, USA) software.

## 3. Results

### 3.1. mRNA Expression in Human Cancers

**Figure 2:**
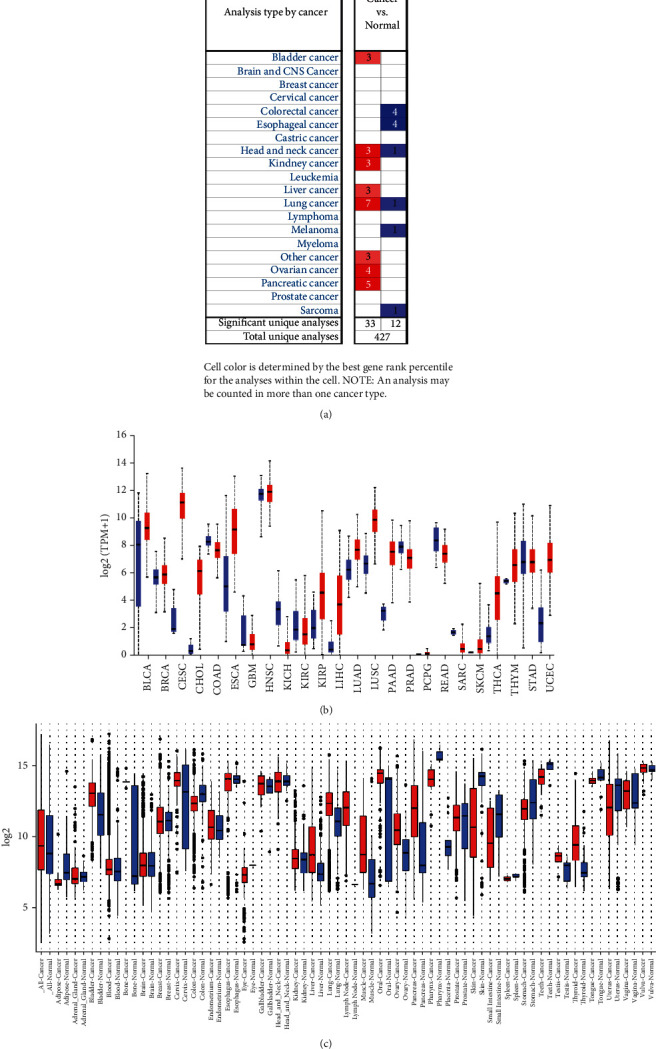
SFN mRNA expression in different types of cancer. (a) A graphic constructed based on the data retrieved from the ONCOMINE database; it indicates the number of statistically significant (*P* < 0.01) datasets. mRNA overexpression is represented in red, and downregulation is represented in blue. (b) Expression across TCGA cancer data; tumor (red) and normal (blue) samples are represented by boxplots. (c) The SFN expression patterns in different cancers were determined by utilizing the GENT2 server, where blue represents healthy cells and red represents cancer cells.

We analyzed the expression pattern of SFN in numerous cancer studies by using the ONCOMINE platform. The results showed that SFN was upregulated in seven cancer types, namely, bladder, head and neck, kidney, liver, lung, ovarian, and pancreatic cancers (Figure 2(a)). In the pancancer view based from the ULCAN tool, we found that SFN was upregulated in 16 cancer types, downregulated in 6 cancer types, and equally expressed in 2 cancer types (Figure 2(b)). We also confirmed the upregulation of SFN in different cancers using the GENT2 tool.

### 3.2. SFN Expression in PC versus Healthy Tissues

**Figure 3:**
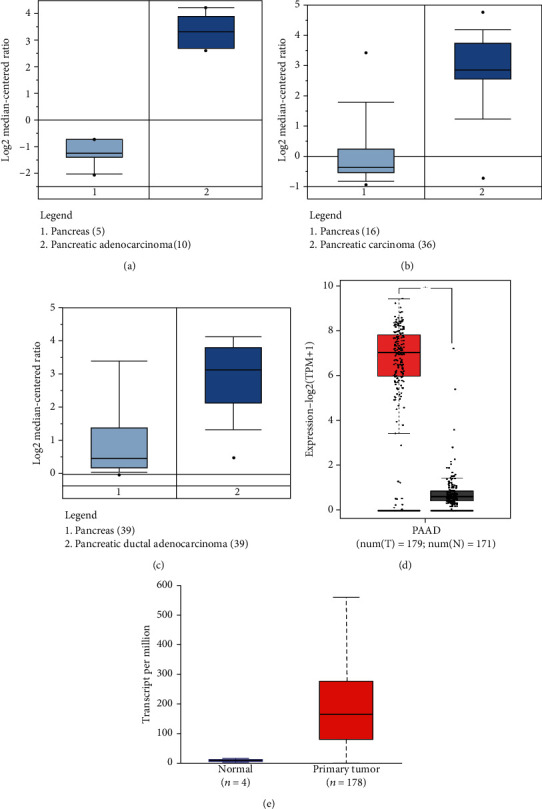
Comparison of SFN expression in PC and normal tissues. (a–c) Boxplot comparing the specific SFN expression in normal (left) and cancer (right) tissues; this boxplot was retrieved from the ONCOMINE tool. (d) Boxplot showing the SFN expression in normal tissue (right) and PC (left) (∗ indicates *P* ≤ 0.05). (e) SFN expression based on the TCGA dataset obtained from UALCAN, where red represents primary tumor and blue represents normal tissues.

SFN was significantly upregulated in different PC types, including pancreatic adenocarcinoma, pancreatic carcinoma, and pancreatic ductal adenocarcinoma, compared with its expression in normal tissues (Figures 3(a)–3(c) and Table 1). Using the GEPIA2 and UALCAN platforms, we further assessed the upregulation of SFN. Our findings indicated that SFN expression was significantly higher in PC tissues than in normal tissues (Figures 3(d) and 3(e)).

### 3.3. SFN Expression in relation to Clinicopathological Parameters in PC

**Figure 4:**
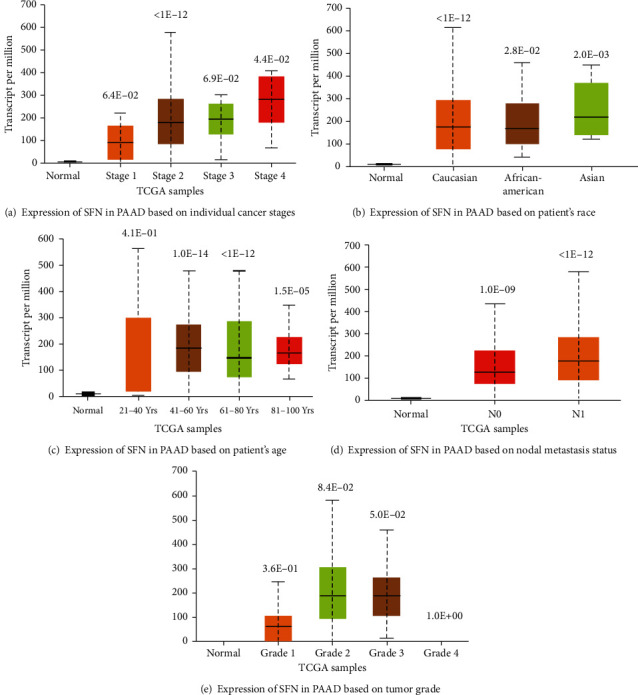
Clinicopathological analysis of SFN in PC: (a) individual cancer stages; (b) patient's race; (c) patient's age; (d) nodal metastasis status; (e) tumor grade.

We looked at variations in SFN gene expression levels in PC patients based on their clinicopathological features. In terms of individual cancer stages, the increase in SFN expression correlated with that in PC progression (Figure 4(a)). In terms of patients' race, SFN expression is increased in Asian patients (Figure 4(b)). In terms of patient's age, higher SFN expression levels were observed in 41–60- and 81–100-year-old patients, whereas lower SFN expression levels were observed in 21–40-year-old patients (Figure 4(c)). As regards nodal metastasis status, a positive nodal status revealed a high SFN expression in PC (Figure 4(d)). Analysis based on tumor grade showed increased SFN expression in grade 3 PC (Figure 4(e)).

### 3.4. Association between SFN Expression and Survival Probability in PC Patients

**Figure 5:**
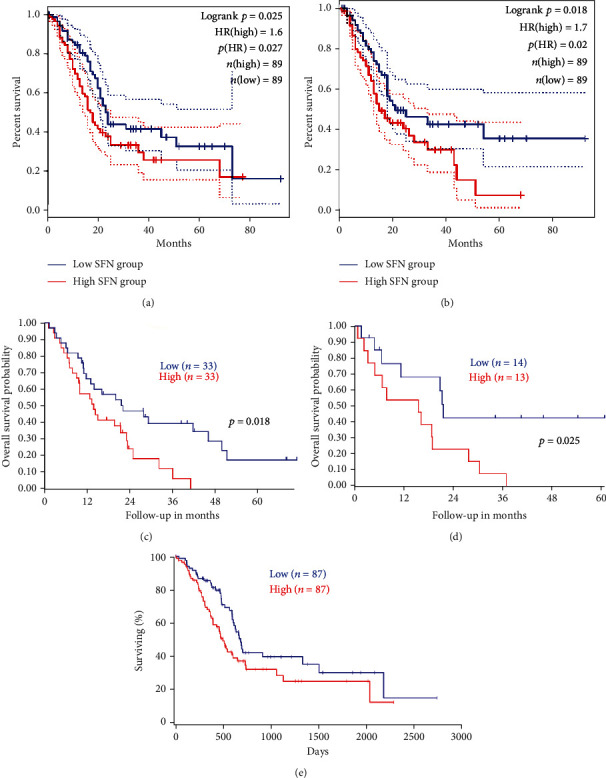
Correlation between SFN expression and prognosis in cancer patients. Red lines indicate SFN overexpression, and blue lines indicate low SFN expression. (a) Overall survival (OS) data retrieved from GEPIA2. (b) Disease-free survival (DFS) data retrieved from GEPIA2. (c) OS from the R2 platform (mixed tumor pancreas Hussain-130-rma-sketch-hugene10t SFN (7899265)). (d) OS from the R2 platform (mixed pancreatic adenocarcinoma Sadanandam-47-MAS5.0-u133p2 SFN (33323-r-at)). (e) OS data collected from the OncoLnc server.

To evaluate the prognostic value of the SFN gene, we determined the survival probability of PC patients using GEPIA2, R2, and OncoLnc. The results obtained from these tools revealed a negative correlation between survival probability and SFN expression (i.e., high SFN expression results in low survival probability). GEPIA2 provided data on the overall and disease-free survival probability of PC patients (Figures 5(a) and 5(b)), whereas R2 and OncoLnc provided information on overall survival probability (Figures 5(c)–5(e)). The analysis results underscored the prognostic relevance of a high SFN expression in PC patients.

### 3.5. Coexpression Analysis of the SFN Gene in PC Cancer

**Figure 6:**
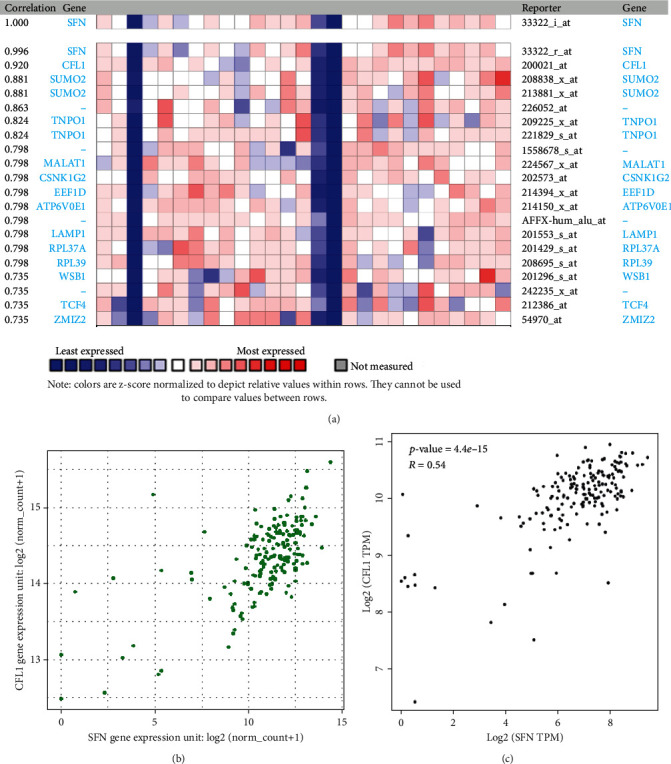
Coexpression analysis of the SFN gene in PC. (a) Heat map presenting the genes that are positively correlated with SFN based on the data retrieved from ONCOMINE. (b) Correlation analysis between SFN and CFL1 using the UCSC Xena web tool. (c) Coexpression of the SFN and CFL1 transcript levels in PC tissue is illustrated using the GEPIA2 web tool.

We determined the genes that are positively associated with SFN expression to identify the coexpressed genes associated with PC development. A heat map (Figure 6(a)) involving 13 genes coexpressed with SFN was obtained from ONCOMINE. Among these genes, CFL1 was strongly (*R* = 0.92) coexpressed with SFN. Moreover, we observed a positive association between SFN and CFL1 using the TCGA data from the UCSC Xena tool, with Pearson's and Spearman's values of 0.67 and 0.60, respectively (Figure 6(b)). The GEPIA2 tool validated the positive correlation between SFN and CFL1, with a Pearson value of 0.54 (Figure 6(c)).

### 3.6. Enrichment Analysis of the SFN Gene

**Figure 7:**
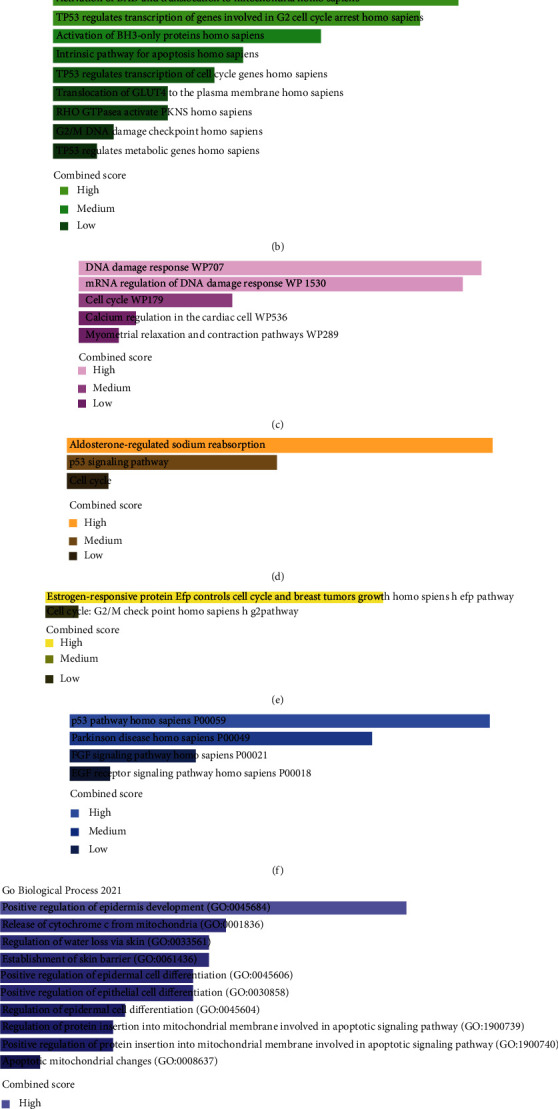
Enrichment analysis of the SFN gene: (a) BioPlanet 2019 pathway; (b) Reactome 2016 pathway; (c) WikiPathway 2021 Human; (d) KEGG 2021 Human pathway; (e) Biocarta 2016 pathway; (f) Panther 2016 pathway; (g) GO Biological Process 2021. (H) GO Molecular Function 2021.

Significantly enriched pathways involving the SFN gene were determined from six databases depicted in Figures 7(a)–7(f). For BioPlanet 2019, we observed significantly enriched pathways, namely, cell cycle control pathway, p38 MK2 pathway, G2/M checkpoint control pathway, insulin regulation of blood glucose, PICK3C/AKT pathway, and PI3K/PLC/TRK pathway (Figure 7(a)). Similarly, Reactome 2016 revealed the significantly enriched pathways related to Chk1/Chk2-mediated inactivation of cyclin B, BAD activation and its translocation to the mitochondria, TP53-regulated G2 cell cycle arrest genes, activation of BH3-only proteins, intrinsic pathway of apoptosis, and TP53-regulated cell genes (Figure 7(b)). In WikiPathway 2021 (Figure 7(c)), KEGG 2021 (Figure 7(d)), Biocarta 2016 (Figure 7(e)), and Panther 2016 (Figure 7(f)), the most prominent pathways were DNA damage response, miRNA regulation of DNA damage response, cell cycle regulation, p53 signaling pathway, and FGF signaling pathway. Furthermore, we investigated the gene ontologies for the SFN gene. The GO Biological Process 2021 determined the predominant biological processes, such as positive regulation of epidermal development, release of cytochrome c from mitochondria, regulation of water loss via the skin, positive regulation of epidermal and epithelial cell differentiation, and apoptotic mitochondrial change (Figure 7(g)). In GO Molecular Function 2021, the most significantly enriched function was the protein serine/threonine kinase inhibitory activity (Figure 7(h)).

### 3.7. Evaluation of the SFN Interaction Network

**Figure 8:**
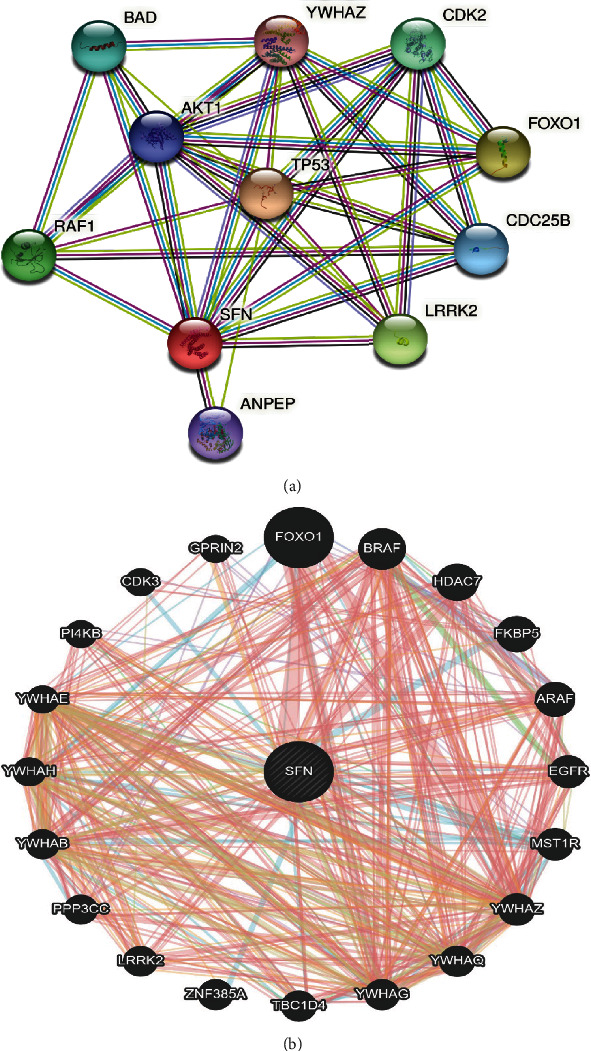
SFN interaction network. SFN-interacting proteins play a role in cell cycle regulation, apoptosis, and cancer. (a) PPI interaction network obtained from the STRING database. (b) SFN interaction network retrieved from GeneMANIA.

We utilized GeneMANIA and STRING, two different web-based network analysis tools, to explore the SFN interaction network. Protein–protein interactions (PPIs) play important roles in cellular activities and biological signaling in all animals, and this information helps researchers to gain a better understanding of various connections and pathways [51]. The PPI network from the STRING database showed the interactions of SFN with TP53, FOXO1, LRRK2, RAF1, CDK2, BAD, CDC25B, AKT1, ANPEP, and YWHAZ (Figure 8(a)). Analysis of the network provided information about the number of nodes (i.e., 11), number of edges (i.e., 49), average node degree (i.e., 8.91), average local clustering coefficient (i.e., 0.922), and PPI enrichment *P* value (i.e., 6.49*e*-10). GeneMANIA revealed the interaction of SNF with FOXO1, BRAF, HDAC7, FKBP5, ARAF, EGFR, MST1R, YWHAZ, YWHAQ, YWHAG, TPC1D4, ZNF385A, LRRK2, PPP3CC, YWHAB, YWHAH, YWHAE, PI4KB, CDK3, and GPRIN2 (Figure 8(b)).

### 3.8. TF and miRNA Network Analyses

**Figure 9:**
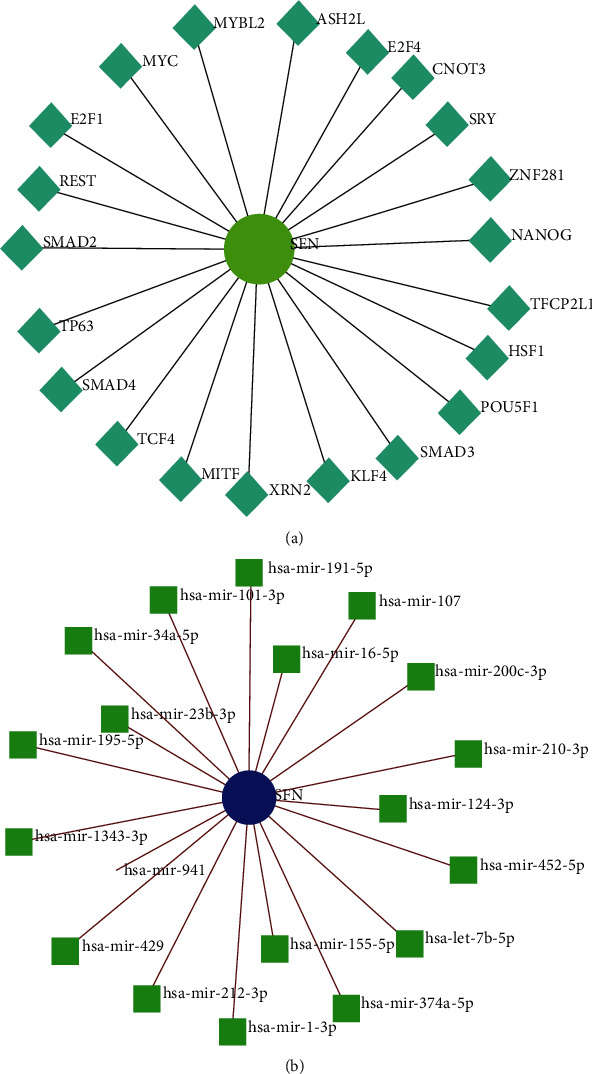
Interaction network of SFN with TFs and miRNAs. (a) TF network constructed from the ChEA database through the NetworkAnalyst platform. (b) miRNA network constructed from the TarBase database using the NetworkAnalyst platform.

The TF network constructed using the NetworkAnalyst platform revealed the direct interaction of 21 transcription factors (TFs) with SFN. The TFs for SFN were ASH2L, E2F4, CNOT3, SRY, ZNF281, NANOG, TFCP2L1, HSF1, POU5F1, SMAD3, KLF4, XRN2, MITF, TCF4, SMAD4, TP63, SMAD2, REST, E2F1, MYC, and MYBL2 (Figure 9(a)). Modification of these TFs might play a significant role in altering the SFN gene expression in PC. In the miRNA analysis, we obtained a network showing the direct interaction of 19 miRNAs with SFN (Figure 9(b)). These miRNAs can modify the SFN expression at the posttranscriptional stage.

### 3.9. ROC Curve Analysis of the SFN Gene

**Figure 10:**
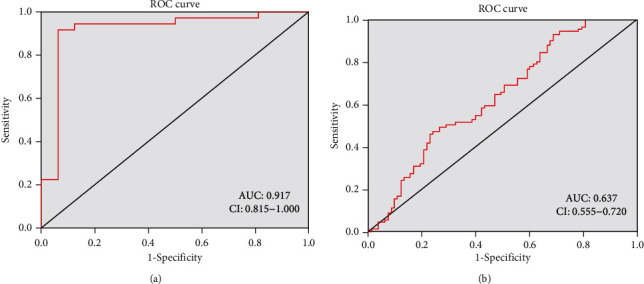
Evaluation of the diagnostic and prognostic values of the SFN gene. (a) ROC curve for SFN expression in pancreatic cancer. (b) ROC curve for patients' survival. AUC: area under the curve, CI: confidence interval.

In the ROC curve, the area under the curve (AUC) is used to discriminate between classes. In the GSE16515 dataset, the AUC for the SFN gene expression was 0.965 (Figure 10(a)) and the AUC for the survival of patients was 0.637 (Figure 10(b)). These AUC results indicate that the SFN gene might be used as a diagnostic and prognostic marker.

## 4. Discussion

PC is one of the most aggressive cancers affecting human health, and it is considered the “silent disease,” as it does not show noticeable symptoms at an early stage [52]. Given that it displays characteristics similar to those of other diseases, such as ulcer, gastritis, and pancreatitis, it is mostly diagnosed at an advanced stage [53]. As early detection remains difficult, finding a potential novel biomarker that aids in early detection is desired. In this study, we utilized bioinformatics approaches to assess the importance of the SFN gene as a biomarker in PC prediction.

Upregulated SFN gene expression in PC and other cancer types was observed in ONCOMINE, UALCAN, and GENT2. The upregulated SFN expression in PC cells was compared with that in normal pancreatic cells using the data from ONCOMINE, GEPIA2, and UALCAN. A study on the molecular profiling of stroma in pancreatic ductal adenocarcinoma has revealed the upregulated expression of SFN, along with other genes [54]. This upregulated SFN expression in PC is supported by other studies [27, 55–57]. Gene expression levels in cancers can vary under different clinicopathological conditions, as cancer is a heterogeneous and complex disease. We analyzed the SFN expression based on patients' age, race, tumor grade, tumor stage, and nodal status. The results showed that SFN was highly upregulated among Asians, among 41–60-year-old individuals, among those with a positive nodal status, and among grade 3 tumor patients. Interestingly, in the case of cancer stages, SFN expression increased proportionally with cancer stage progression. Then, the prognostic value of SFN in PC was evaluated using the GEPIA2, OncoLnc, and R2 platforms. High SFN expression significantly (*P* < 0.05) correlated with low overall and disease-free survival. Our current findings agreed with those of another study in which SFN was considered an independent prognostic biomarker in pancreatic ductal adenocarcinoma [54]. Moreover, it has been demonstrated that the elevated 14-3-3*σ* protein levels likely contribute to the poor prognostic outcome of human pancreatic tumors, as they promote resistance to radiation and anticancer treatments [15].

Gene coexpression provides information that aids in the identification of functionally linked genes. Coexpression analysis using the ONCOMINE platform revealed 13 genes, among which CFL1 was highly coexpressed with SFN. Furthermore, CFL1 coexpression in PC was confirmed by GEPIA2 and UCSC Xena. CFL1 is a small, ubiquitous, actin-binding protein that plays important roles in cytokinesis, endocytosis, apoptosis, cell proliferation, and migration, as well as in tumor development, infiltration, and metastasis [58, 59]. Moreover, it has been reported that this protein is necessary for the invasion and spread of numerous human malignant solid tumors [60, 61]. Recent studies have found a positive association between high CFL1 gene expression and PC progression [59, 62].

Enrichment analysis for the SFN gene was performed by utilizing the Enrichr web platform. The most prominent pathways, including cell cycle control, Chk1/Chk2-mediated inactivation of cyclin B, DNA damage response, aldosterone-regulated sodium reabsorption, estrogen-responsive protein efp control cell cycle, and p53 pathway, were obtained from BioPlanet 2019, Reactome 2016, WikiPathway 2021, KEGG 2021, Biocarta 2016, and Panther 2016, respectively. SFN was initially found to be a p53-inducible gene that responds to DNA-damaging agents [63]. A study has reported that SFN inhibits the initiation of mitosis by sequestering the mitotic initiation complex (cdc2-cyclin B1) and preventing it from entering the nucleus [64]. In this manner, SFN causes G2 arrest, allowing damaged DNA to be repaired. It has been demonstrated that SFN directly controls the G2/M checkpoint of the cell cycle by protecting p53 against MDM2-mediated ubiquitination and degradation [65–67]. These findings indicate that SFN acts as a negative regulator of cell cycle progression and might be considered a tumor suppressor. However, SFN plays a double-edged function in human cancers, and its function may vary among organs and tissues [32, 68]. Meanwhile, accumulation of 14-3-3*σ* has been observed in PC, but it cannot perform its major ascribed functions, such as sustaining a G2 checkpoint and performing an antiapoptotic action, due to multiple alterations in its interaction with downstream partners [69].

Network analysis based from the STRING database revealed the functional interaction partners of SFN, namely, TP53, FOXO1, LRRK2, RAF1, CDK2, BAD, CDC25B, AKT1, ANPEP, and YWHAZ. It has been reported that overexpression of CDC25B is associated with pancreatic ductal adenocarcinoma and that its inhibitor prevents PC cell growth by blocking the G2/M phase transition via the inhibition of cdc2 dephosphorylation [70]. According to the NCBI, defects in the ANPEP gene enhances angiogenesis, tumor growth, and metastasis [71]. Meanwhile, overexpression of the YWHAZ gene has been demonstrated to be a prognostic and therapeutic target in gastric cancer [72, 73]. In GeneMANIA, SFN shares consolidated pathways with MST1R and YWHAG. MST1R expression has been shown to play an oncogenic function in human pancreatic intraepithelial neoplasia, as well as in primary human and animal metastatic cell lines [74]. In PC, the overexpression of the YWHAG gene is associated with poor overall survival compared with low YWHAG expression [75]. Furthermore, our network analysis revealed some TFs and miRNAs that might play important roles in determining how SFN gene expression is regulated at the transcriptional and posttranscriptional levels.

In ROC analysis, SFN expression showed excellent (AUC = 0.917) diagnostic value of pancreatic cancer. A meta-analysis study showed that the sensitivity and specificity of CA 19-9 were 78.2% and 82.8%, respectively [76]. However, the CA 19-9 level may be augmented in other medical conditions, such as acute cholangitis, pancreatitis, obstructive jaundice, and liver cirrhosis [11]. In our study, SFN also exhibited as a good (AUC = 0.637) prognostic marker in pancreatic cancer. In these aspects, SFN might be considered as an auxiliary biomarker of CA 19-9 in PC. Of course, there are some limitations in our study. First, due to the lack of enough datasets, the sample size for analysis was relatively small. Second, the absence of in vivo and in vitro experiments is another flaw of our study. Third, this study cannot explain how the tissue-specific upregulation SFN gene is related to pancreatic cancer. Therefore, further wet laboratory molecular studies are needed.

## 5. Conclusion

Data from the online bioinformatics platforms utilized in this study showed that SFN expression in PC was upregulated relative to that in normal tissues. Moreover, a negative correlation between SFN expression and survival probability was found in PC. In our network analysis, SFN-associated proteins, TFs, and miRNAs were identified. Based on these findings, we can conclude that the high tissue-dependent SFN expression might be used as a biomarker for diagnosis, prognosis, and therapeutic purposes. However, further wet laboratory-based studies are needed to bolster the significance of SFN overexpression in PC.

## Figures and Tables

**Table 1:** SFN expression in different PC subtypes.

Datasets	Pancreatic cancer subtype	*P* value	*t* test	Fold change
Logsdon pancreas	Pancreatic adenocarcinoma (10)	7.02*E*-9	16.428	24.921
Iacobuzio-Donahue pancreas 2	Pancreatic adenocarcinoma (12)	1.14*E*-6	10.684	20.186
Segara pancreas	Pancreatic carcinoma (11)	3.31*E*-6	7.810	9.735
Pei pancreas	Pancreatic carcinoma (36)	2.27*E*-9	8.043	7.017
Badea pancreas	Pancreatic ductal adenocarcinoma (39)	1.82*E*-11	7.746	6.660

## Data Availability

Any data or information used in this current study is available from the corresponding author on reasonable request.

## References

[1] Bray F., Ferlay J., Soerjomataram I., Siegel R. L., Torre L. A., Jemal A. (2018). Global cancer statistics 2018: GLOBOCAN estimates of incidence and mortality worldwide for 36 cancers in 185 countries. *CA: a Cancer Journal for Clinicians*.

[2] Rawla P., Sunkara T., Gaduputi V. (2019). Epidemiology of pancreatic cancer: global trends, etiology and risk factors, world. *World journal of oncology*.

[3] Siegel R. L., Miller K. D., Jemal A. (2019). Cancer statistics, 2019. *Cancer Journal for Clinicians*.

[4] Khalaf N., El-Serag H. B., Abrams H. R., Thrift A. P. (2021). Burden of pancreatic cancer: from epidemiology to practice. *Clinical Gastroenterology and Hepatology*.

[5] (2021). Pancreatic Cancer: Statistics | Cancer.Net. https://www.cancer.net/cancer-types/pancreatic-cancer/statistics.

[6] Ferlay J., Partensky C., Bray F. (2016). More deaths from pancreatic cancer than breast cancer in the EU by 2017. *Acta Oncologica*.

[7] Al-Shaheri F. N., Alhamdani M. S. S., Bauer A. S. (2021). Blood biomarkers for differential diagnosis and early detection of pancreatic cancer. *Cancer Treatment Reviews*.

[8] Turanli B., Yildirim E., Gulfidan G., Arga K. Y., Sinha R. (2021). Current state of “Omics” biomarkers in pancreatic cancer. *J. Pers. Med.*.

[9] Siegel R. L., Miller K. D., Jemal A. (2018). Cancer statistics, 2018. *Cancer Journal for Clinicians*.

[10] Quaresma M., Coleman M. P., Rachet B. (2015). 40-year trends in an index of survival for all cancers combined and survival adjusted for age and sex for each cancer in England and Wales, 1971-2011: a population-based study. *Lancet*.

[11] Hasan S., Jacob R., Manne U., Paluri R. (2019). Advances in pancreatic cancer biomarkers. *Oncology Reviews*.

[12] Goonetilleke K. S., Siriwardena A. K. (2007). Systematic review of carbohydrate antigen (CA 19-9) as a biochemical marker in the diagnosis of pancreatic cancer. *European Journal of Surgical Oncology*.

[13] Ballehaninna U. K., Chamberlain R. S. (2012). The clinical utility of serum CA 19-9 in the diagnosis, prognosis and management of pancreatic adenocarcinoma: an evidence based appraisal. *J. Gastrointest. Oncol.*.

[14] Poruk K. E., Firpo M. A., Adler D. G., Mulvihill S. J. (2013). Screening for pancreatic cancer: why, how, and who?. *Annals of Surgery*.

[15] Li Z., Dong Z., Myer D. (2010). Role of 14-3-3*σ* in poor prognosis and in radiation and drug resistance of human pancreatic cancers. *BMC Cancer*.

[16] Prasad G. L., Valverius E. M., McDuffie E., Cooper H. L. (1992). Complementary DNA cloning of a novel epithelial cell marker protein, HME1, that may be down-regulated in neoplastic mammary cells. *Cell Growth & Differentiation*.

[17] Laronga C., Yang H. Y., Neal C., Lee M. H. (2000). Association of the cyclin-dependent kinases and 14-3-3 sigma negatively regulates cell cycle progression∗. *The Journal of Biological Chemistry*.

[18] Vercoutter-Edouart A. S., Lemoine J., Le Bourhis X. (2001). Proteomic analysis reveals that 14-3-3sigma is down-regulated in human breast cancer cells. *Cancer Research*.

[19] Osada H., Tatematsu Y., Yatabe Y. (2002). Frequent and histological type-specific inactivation of 14-3-3*σ* in human lung cancers. *Oncogene*.

[20] Iwata N., Yamamoto H., Sasaki S. (2000). Frequent hypermethylation of CpG islands and loss of expression of the ∗∗_14-3-3_∗∗*σ* gene in human hepatocellular carcinoma. *Oncogene*.

[21] Nakayama H., Sano T., Motegi A., Oyama T., Nakajima T. (2005). Increasing 14-3-3 sigma expression with declining estrogen receptor *α* and estrogen-responsive finger protein expression defines malignant progression of endometrial carcinoma. *Pathology International*.

[22] Gasco M., Bell A. K., Heath V. (2002). Epigenetic inactivation of 14-3-3 sigma in oral carcinoma: association with p16(INK4a) silencing and human papillomavirus negativity. *Cancer Research*.

[23] Uchida D., Begum N. M., Almofti A., Kawamata H., Yoshida H., Sato M. (2004). Frequent downregulation of 14-3-3 _*σ*_ protein and hypermethylation of _14-3-3 *σ*_ gene in salivary gland adenoid cystic carcinoma. *British Journal of Cancer*.

[24] Gasco M., Sullivan A., Repellin C. (2002). Coincident inactivation of 14-3-3*σ* and p16^INK4a^ is an early event in vulval squamous neoplasia. *Oncogene*.

[25] Lodygin D., Diebold J., Hermeking H. (2004). Prostate cancer is characterized by epigenetic silencing of _14-3-3 *σ*_ expression. *Oncogene*.

[26] Cheng L., Pan C. X., Zhang J. T. (2004). Loss of 14-3-3*σ* in prostate cancer and its precursors. *Clinical Cancer Research*.

[27] Logsdon C. D., Simeone D. M., Binkley C. (2003). Molecular profiling of pancreatic adenocarcinoma and chronic pancreatitis identifies multiple genes differentially regulated in pancreatic cancer. *Cancer Research*.

[28] Friess H., Ding J., Kleeff J. (2003). Microarray-based identification of differentially expressed growth- and metastasis-associated genes in pancreatic cancer. *Cellular and Molecular Life Sciences*.

[29] Iacobuzio-Donahue C. A., Ashfaq R., Maitra A. (2003). Highly expressed genes in pancreatic ductal adenocarcinomas: a comprehensive characterization and comparison of the transcription profiles obtained from three major technologies. *Cancer Research*.

[30] Perathoner A., Pirkebner D., Brandacher G. (2005). 14-3-3*Σ* expression is an independent prognostic parameter for poor survival in colorectal carcinoma patients. *Clinical Cancer Research*.

[31] Okumura H., Kita Y., Yokomakura N. (2010). Nuclear expression of 14-3-3 sigma is related to prognosis in patients with esophageal squamous cell carcinoma. *Anticancer Research*.

[32] Li Z., Liu J. Y., Zhang J. T. (2009). 14-3-3sigma, the double-edged sword of human cancers. *American Journal of Translational Research*.

[33] Rhodes D. R., Kalyana-Sundaram S., Mahavisno V. (2007). Oncomine 3.0: genes, pathways, and networks in a collection of 18,000 cancer gene expression profiles. *Neoplasia*.

[34] Rhodes D. R., Yu J., Shanker K. (2004). _ONCOMINE : a cancer microarray database and integrated data-mining platform. *Neoplasia*.

[35] Chandrashekar D. S., Bashel B., Balasubramanya S. A. H. (2017). UALCAN: a portal for facilitating tumor subgroup gene expression and survival analyses. *Neoplasia (United States)*.

[36] Park S. J., Yoon B. H., Kim S. K., Kim S. Y. (2019). GENT2: an updated gene expression database for normal and tumor tissues. *BMC Medical Genomics*.

[37] Tang Z., Kang B., Li C., Chen T., Zhang Z. (2019). GEPIA2: an enhanced web server for large-scale expression profiling and interactive analysis. *Nucleic Acids Research*.

[38] (2021). R2: Kaplan-Meier. https://hgserver1.amc.nl/cgi-bin/r2/main.cgi?option=kaplan_main.

[39] Anaya J. (2016). OncoLnc: linking TCGA survival data to mRNAs, miRNAs, and lncRNAs. *PeerJ Computer Science*.

[40] Goldman M. J., Craft B., Hastie M. (2020). Visualizing and interpreting cancer genomics data via the Xena platform. *Nature Biotechnology*.

[41] Ginestet C. (2011). Eggplot2: elegant graphics for data analysis. *J. R. Stat. Soc. Ser. A Statistics Soc*.

[42] Chen E. Y., Tan C. M., Kou Y. (2013). Enrichr: interactive and collaborative HTML5 gene list enrichment analysis tool. *BMC Bioinformatics*.

[43] Kuleshov M. V., Jones M. R., Rouillard A. D. (2016). Enrichr: a comprehensive gene set enrichment analysis web server 2016 update. *Nucleic Acids Research*.

[44] Szklarczyk D., Morris J. H., Cook H. (2017). The STRING database in 2017: quality-controlled protein-protein association networks, made broadly accessible. *Nucleic Acids Research*.

[45] Warde-Farley D., Donaldson S. L., Comes O. (2010). The GeneMANIA prediction server: biological network integration for gene prioritization and predicting gene function. *Nucleic Acids Research*.

[46] Mitsis T., Efthimiadou A., Bacopoulou F., Vlachakis D., Chrousos G. P., Eliopoulos E. (2020). Transcription factors and evolution: an integral part of gene expression (review). *World Academy of Sciences Journal*.

[47] O’Brien J., Hayder H., Zayed Y., Peng C. (2018). Overview of microRNA biogenesis, mechanisms of actions, and circulation. *Frontiers in endocrinology*.

[48] Lachmann A., Xu H., Krishnan J., Berger S. I., Mazloom A. R., Ma'ayan A. (2010). ChEA: transcription factor regulation inferred from integrating genome-wide ChIP-X experiments. *Bioinformatics*.

[49] Karagkouni D., Paraskevopoulou M. D., Chatzopoulos S. (2018). DIANA-TarBase v8: a decade-long collection of experimentally supported miRNA-gene interactions. *Nucleic Acids Research*.

[50] Xia J., Gill E. E., Hancock R. E. W. (2015). NetworkAnalyst for statistical, visual and network-based meta-analysis of gene expression data. *Nature Protocols*.

[51] de Las Rivas J., Fontanillo C. (2010). Protein-protein interactions essentials: key concepts to building and analyzing interactome networks. *PLoS Computational Biology*.

[52] Sheth N., Dalbagni G., Rothenberg R. E., LaRaja R. D. (1985). Carcinoma of the pancreas in nonjaundiced patients. A silent disease. *The American Surgeon*.

[53] Khan T., Paul B. K., Hasan M. T. (2021). Significant pathway and biomarker identification of pancreatic cancer associated lung cancer. *Informatics in Medicine Unlocked*.

[54] Robin F., Angenard G., Cano L. (2020). Molecular profiling of stroma highlights stratifin as a novel biomarker of poor prognosis in pancreatic ductal adenocarcinoma. *British Journal of Cancer*.

[55] Okada T., Masuda N., Fukai Y. (2006). Immunohistochemical expression of 14-3-3 sigma protein in intraductal papillary-mucinous tumor and invasive ductal carcinoma of the pancreas. *Anticancer Research*.

[56] Neupane D., Korc M. (2008). 14-3-3*σ* modulates pancreatic cancer cell survival and invasiveness. *Clinical Cancer Research*.

[57] Iacobuzio-Donahue C. A., Maitra A., Olsen M. (2003). Exploration of global gene expression patterns in pancreatic adenocarcinoma using cDNA microarrays. *The American Journal of Pathology*.

[58] Mousavi S., Safaralizadeh R., Hosseinpour-Feizi M., Azimzadeh-Isfanjani A., Hashemzadeh S. (2018). Study of cofilin 1 gene expression in colorectal cancer. *Journal of Gastrointestinal Oncology*.

[59] Wang L., Xiong L., Wu Z. (2018). Expression of UGP2 and CFL1 expression levels in benign and malignant pancreatic lesions and their clinicopathological significance. *World Journal of Surgical Oncology*.

[60] Klamt F., Zdanov S., Levine R. L. (2009). Oxidant-induced apoptosis is mediated by oxidation of the actin-regulatory protein cofilin. *Nature Cell Biology*.

[61] Wang W., Mouneimne G., Sidani M. (2006). The activity status of cofilin is directly related to invasion, intravasation, and metastasis of mammary tumors. *The Journal of Cell Biology*.

[62] Werle S. D., Schwab J. D., Tatura M. (2021). Unraveling the molecular tumor-promoting regulation of cofilin-1 in pancreatic cancer. *Cancers (Basel).*.

[63] Hermeking H., Lengauer C., Polyak K. (1997). _∗∗14-3-3 *σ*∗∗_ ∗∗is a p53-regulated inhibitor of G2/M progression∗∗. *Molecular Cell*.

[64] Chan T. A., Hermeking H., Lengauer C., Kinzler K. W., Vogelstein B. (1999). 14-3-3*σ* is required to prevent mitotic catastrophe after DNA damage. *Nature*.

[65] Yang H., Zhao R., Lee M. H. (2006). 14-3-3*Σ*, a P53 regulator, suppresses tumor growth of nasopharyngeal carcinoma. *Molecular Cancer Therapeutics*.

[66] Yang H.-Y., Wen Y.-Y., Chen C.-H., Lozano G., Lee M.-H. (2003). 14-3-3*σ* positively regulates p53 and suppresses tumor growth. *Molecular and Cellular Biology*.

[67] West-Foyle H., Kothari P., Osborne J., Robinson D. N. (2018). 14-3-3 proteins tune non-muscle myosin II assembly. *The Journal of Biological Chemistry*.

[68] Shiba-Ishii A., Kano J., Morishita Y., Sato Y., Minami Y., Noguchi M. (2011). High expression of stratifin is a universal abnormality during the course of malignant progression of early-stage lung adenocarcinoma. *International Journal of Cancer*.

[69] Guweidhi A., Kleeff J., Giese N. (2004). Enhanced expression of 14-3-3sigma in pancreatic cancer and its role in cell cycle regulation and apoptosis. *Carcinogenesis*.

[70] Guo J., Kleeff J., Li J. (2004). Expression and functional significance of CDC25B in human pancreatic ductal adenocarcinoma. *Oncogene*.

[71] (2021). ANPEP alanyl aminopeptidase, membrane [Homo sapiens (human)] - gene - NCBI. https://www.ncbi.nlm.nih.gov/gene/290.

[72] Nishimura Y., Komatsu S., Ichikawa D. (2013). Overexpression of YWHAZ relates to tumor cell proliferation and malignant outcome of gastric carcinoma. *British Journal of Cancer*.

[73] Watanabe N., Komatsu S., Ichikawa D. (2016). Overexpression of YWHAZ as an independent prognostic factor in adenocarcinoma of the esophago-gastric junction. *American Journal of Cancer Research*.

[74] Thomas R. M., Toney K., Fenoglio-Preiser C. (2007). The RON receptor tyrosine kinase mediates oncogenic phenotypes in pancreatic cancer cells and is increasingly expressed during pancreatic cancer progression. *Cancer Research*.

[75] Liu P., Kong L., Liang K. (2020). Identification of dissociation factors in pancreatic cancer using a mass spectrometry-based proteomic approach. *BMC Cancer*.

[76] Poruk K. E., Gay D. Z., Brown K. (2013). The clinical utility of CA 19-9 in pancreatic adenocarcinoma: diagnostic and prognostic updates. *Current Molecular Medicine*.

